# Stereoselectivity of Electron and Energy Transfer in the Quenching of (*S*/*R*)-Ketoprofen-(*S*)-Tryptophan Dyad Excited State

**DOI:** 10.3390/ijms21155370

**Published:** 2020-07-28

**Authors:** Aleksandra A. Ageeva, Simon V. Babenko, Ilya M. Magin, Victor F. Plyusnin, Polina S. Kuznetsova, Alexander A. Stepanov, Sergey F. Vasilevsky, Nikolay E. Polyakov, Alexander B. Doktorov, Tatyana V. Leshina

**Affiliations:** 1 Voevodsky Institute of Chemical Kinetics and Combustion SB RAS, 630090 Novosibirsk, Russia; simonb683@gmail.com (S.V.B.); magin@kinetics.nsc.ru (I.M.M.); plyusnin@kinetics.nsc.ru (V.F.P.); Pol0596@yandex.ru (P.S.K.); stepanov@kinetics.nsc.ru (A.A.S.); vasilev@kinetics.nsc.ru (S.F.V.); polyakov@kinetics.nsc.ru (N.E.P.); doktorov@kinetics.nsc.ru (A.B.D.); leshina@ngs.ru (T.V.L.); 2 Department of Natural Sciences, Novosibirsk State University, 630090 Novosibirsk, Russia

**Keywords:** chiral linked systems, diastereomers, electron transfer, resonance energy transfer, fluorescence, stereoselectivity

## Abstract

Photoinduced elementary processes in chiral linked systems, consisting of drugs and tryptophan (Trp) residues, attract considerable attention due to several aspects. First of all, these are models that allow one to trace the full and partial charge transfer underlying the binding of drugs to enzymes and receptors. On the other hand, Trp fluorescence is widely used to establish the structure and conformational mobility of proteins due to its high sensitivity to the microenvironment. Therefore, the study of mechanisms of Trp fluorescence quenching in various systems has both fundamental and practical interest. An analysis of the photo-chemically induced dynamic nuclear polarization (CIDNP) and Trp fluorescence quenching in (*R*/*S*)-ketoprofen-(*S*)-tryptophan ((*S*/*R*)-KP-(*S*)-Trp) dyad carried out in this work allowed us to trace the intramolecular reversible electron transfer (ET) and obtain evidence in favor of the resonance energy transfer (RET). The fraction of dyad’s singlet excited state, quenched via ET, was shown to be 7.5 times greater for the (*S*,*S*)-diastereomer than for the (*R*,*S*) analog. At the same time, the ratio of the fluorescence quantum yields shows that quenching effectiveness of (*S*,*S*)-diastereomer to be 5.4 times lower than for the (*R*,*S*) analog. It means that the main mechanism of Trp fluorescence quenching in (*S*/*R*)-KP-(*S*)-Trp dyad is RET.

## 1. Introduction

The electron transfer (ET) in chemical and biological processes has long been of great interest to researchers [1,2]. According to the modern point of view, ET in living systems is involved in several vital processes: photosynthesis, functioning of several enzymes, as well as pathological processes. The last is oxidative stress, including DNA damage known to underlie several diseases and aging. 

In recent decades, studies of photoinduced ET in linked systems—dyads with two chiral biologically active partners (drugs, amino acids)—have attracted considerable attention [3,4,5,6,7]. This interest is due to the fact that such dyads are considered as a model to study the mechanisms of drugs binding with enzymes and receptors. Although all active sites include chiral amino acid residues, the model is relevant when the interaction between drugs and protein involves stages with charge transfer [7]. Therefore, there is a point of view that upon the binding of chiral drug with the amino acids located in the active site of the enzymes or receptors, an analogue of diastereomer is formed [4,5,6,7]. 

**Figure 1:**
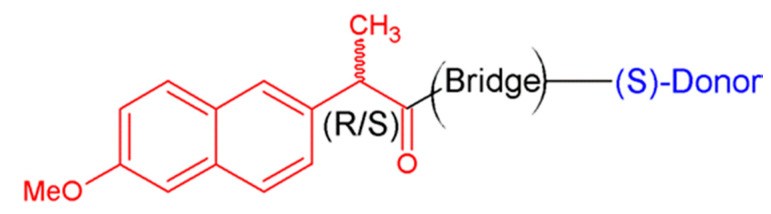
Dyads including NSAID naproxen (NPX, red). Donors: N-methylpyrrolidine, tryptophan.

The main result of the studies of dyads, involving chiral drug residues, is the appearance of the difference of the photoinduced ET rates for diastereomers, so-called stereodifferentiation. This finding can shed light on the chemical nature of differences in the medicinal activity of drug enantiomers [6,7]. Note that the nature of these differences is a practical problem of medicinal chemistry and pharmacology [7]. In addition, most of the studied dyads that demonstrated difference in the rates of photoinduced ET included non-steroidal anti-inflammatory drugs (NSAIDs) (Figure 1). Representatives of this class of drugs are known to show significant differences in the medicinal activity of enantiomers [6,7,8,9,10,11]. 

Besides the stereodifferentiation, photoinduced intramolecular reversible ET in diastereomers has demonstrated another peculiarity. This is the difference in the efficiency of back ET in intermediates—biradical zwitterions [12,13]. Efficiency is defined as spin selectivity: the difference between the chemically induced dynamic nuclear polarization (CIDNP) enhancement coefficients of protons in a dyad’s diastereomers [12,13].

Because the CIDNP has played a significant role in detecting electron transfer stages in various chemical and biochemical processes, and it is the main research method in this article, we will touch a little on its achievements and opportunities for ET studies [14,15,16]. Chemical polarization of nuclei is the appearance in the NMR spectra of the products of radical reactions occurring in the probe of the spectrometer, the signals of which have a non-Boltzmann population of nuclear spin states (hyperpolarization). Hyperpolarization arises as a result of differences in the rates of singlet–triplet conversion for different projections of nuclear spins, which in turn is a consequence of an electron-nuclear interaction in a pair of radicals. Thus, analysis of the CIDNP effects of individual groups of nuclei in the NMR spectra allows one to obtain “the portrait” of a radical pair—the precursor of polarized products—and to establish the role of the radical stages in the process under study. The CIDNP method has been described in detail elsewhere, and is part of the phenomenon known as “spin chemistry” [14]. Spin chemistry studies the radical reactions in which the rate, and often the direction of the process, is dependent on the electron-nuclear interaction in paramagnetic product precursors and the external magnetic field strength [14].

Over the half a century of its existence, spin chemistry has turned out to be one of the most informative indirect methods for identifying the ET stage in various chemical and biochemical processes [14,15,16]. Among these is the excited states quenching of aromatic compounds by electron donors or acceptors, including the ET mechanism of photoinduced cis-trans isomerization of stilbenes, substituted polyenes—organic pigments and all trans-retinal—polyene chromophore, which is the chemical basis for vertebrate vision [14]. It is significant that before the discovery of CIDNP, these processes were associated with energy transfer.

The analyses of CIDNP data, as well as the results of the study of the influence of magnetic field on reaction rates, allow one to establish detailed schemes for the single-electron oxidation of nicotinamide adenine dinucleotide and its analogues of dihydropyridines, occurring in enzymatic oxidation by horseradish peroxidase [15]. These methods show the role of ET in the action of potential anti-cancer agents—quinones-chelators [16].

The important CIDNP peculiarity is the high sensitivity of hyperpolarization to the changes of spin and molecular dynamics in radical intermediates. In the case of chiral systems, the analysis of the difference in spin dynamics of paramagnetic forms of diastereomers, formed as a result of photoinduced ET, has provided new data on chiral drugs reactivity [12,13]. The main result of the application of CIDNP to chiral systems is the conclusion about the difference between hyperfine coupling constants of paramagnetic forms of diastereomers that also suggest a difference in the distribution of electron densities. The latter is directly related to the reactivity of the enantiomers of NSAID naproxen being a part of diastereomers (Figure 1).

**Figure 2:**
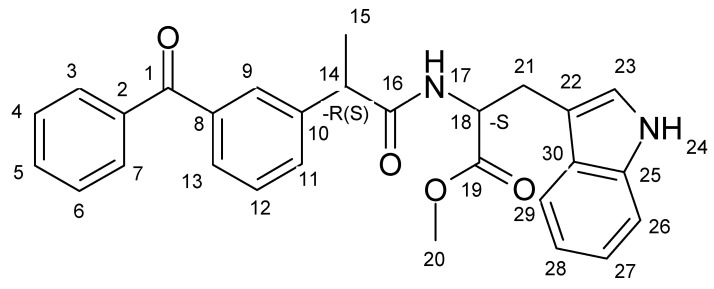
(*S/R*)-ketoprofen-(*S*)-tryptophan ((*S/R*)-KP-(*S*)-Trp) dyad.

This article is also devoted to the study of photoinduced ET between another NSAID representative, ketoprofen, and Trp residue in the (*S/R*)-ketoprofen-(*S*)-tryptophan ((*S/R*)-KP-(*S*)-Trp) dyad (Figure 2).

To date, only indirect data on ET in systems with ketoprofen are available [17]. Meanwhile, the stereo differentiation of another elementary process—photoinduced hydrogen atom transfer in the dyads (*S*)-ketoprofen-(*S/R*)-tetrahydrofuran and (*S*/*R*)-ketoprofen-(*S*)-N-methylpyrrolidine—has been demonstrated [3,17].

The study of photoinduced interactions in the (*S/R*)-KP-(*S*)-Trp dyad by CIDNP and fluorescence technique provides the opportunity to trace both mechanisms of Trp fluorescence quenching: ET and resonance energy transfer (RET). Trp fluorescence is intensively studied today, due to the strong sensitivity of intensity to Trp being surrounded by protein [18,19,20,21]. It is commonly used to identify a variety of protein changes, e.g., ligand-substrate binding, folding-unfolding processes, etc. In particular, Trp fluorescence analysis helps with the diagnosis of the state of the lens of the eye, the study of abnormalities in the processes of protein folding, the study of the stereospecific activity of quantum dots with D and L-amino acids [18,19,20,21]. It is currently believed that the aging of the eye lens and protein folding are accompanied by changes in the optical configuration (chiral inversion) of amino acids; first of all is the transformation of L-Trp to D-isomer. However, the properties of D and L-Trp that lead to changes in the properties of proteins—for example, that influence the folding—remain unknown [19,20,21]. In this regard, it is considered relevant to study the activity of enantiomers using examples of elementary processes [21].

Therefore, in the present work, the comparison of CIDNP data and fluorescence quenching results is used to establish the role of ET and RET in quenching the (*S*)-Trp excited state by (*S*)- and (*R*)-ketoprofen and to trace the difference between the (*S,S*) and (*R,S*)-diastereomers of dyad.

## 2. Results and Discussion

### 2.1. CIDNP Study

The studied (*S/R*)-KP-(*S*)-Trp dyad consists of two chromophores that absorb light at 308 nm. According to reference data, two mechanisms of quenching Trp excited states in biomolecules and donor-acceptor dyads are possible: ET and RET [22,23,24,25]. Parameters confirming the possibility of the presence of these two mechanisms in the (*S/R*)-KP-(*S*)-Trp dyad are listed in Table 1. It is apparent from this Table that transfer of energy would be thermodynamically allowed from the Trp, being in singlet and triplet excited states, to KP in its ground state [26].

As for the processes with charge transfer, in accordance with the Rehm–Weller–Zachariasse criterion, it will be favorable if the free energy (Δ*G_ET_*, Δ*G_exc_*) changes are negative [27]:

(1) $$\Delta G_{ET}=E_{ox}-E_{red}-E_{00}+\frac{2.6\;eV}{\varepsilon }-0.13\;eV,$$

(2) $$\Delta G_{exc}=E_{ox}-E_{red}-E_{00}-\frac{\mu^{2}}{\rho^{3}}\left(\frac{\varepsilon -1}{2\varepsilon +1}-0.19\right)+0.38\;eV$$

where *E_ox_*—the half-wave polarographic oxidation potential of the donor molecule, *E_red_*—the half-wave polarographic reduction potential of the acceptor molecule, *E*_00_—molecular zero–zero transition energy, *μ*^2^/*ρ*^3^—the energy value characterizing the solvation free energy of the dipolar exciplex (with dipole moment μ and equivalent sphere radius *ρ* according to the Kirkwood–Onsager model), *ε*—solvent polarity (=dielectric constant).

**Figure 3:**
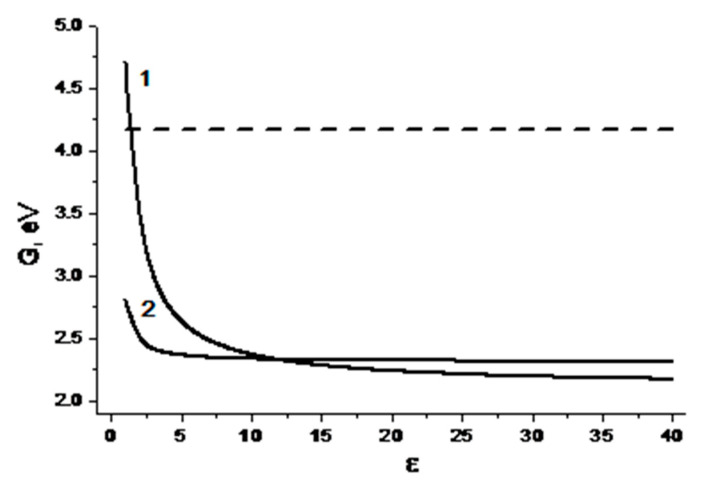
Dependences of the free energy of (*S/R*)-KP-(*S*)-Trp biradical zwitterion on dielectric constant of the solvent (ε). Dashed line shows the energy of an excited singlet state of Trp; 1—biradical zwitterion, 2—exciplex formation.

The curves in Figure 3, obtained using Equations (1) and (2), show that quenching of Trp fluorescence via the processes with charge transfer can be expected in polar media. It might be full (ET) or partial charge transfer (exciplex formation) [27,28].

To study charge transfer processes in (*S,S*) and (*R,S*)-diastereomers, the CIDNP technique was used. Two series of experiments under UV irradiation on (*S*)-KP-(*S*)-Trp and mixtures of diastereomers containing 53% of (*S,S*) and 47% of (*R,S*) were performed in the probe of NMR spectrometer. Since the chemical shifts for the (*R,S*) and (*S,S*) configurations are different for the following protons—NH, CH_2_ of Trp residue, and CH_3_ of KP fragment—it is possible to detect transformation of (*R,S*)- and (*S,S*)-diastereomers by NMR and CIDNP spectra of their mixture (see Figures S1–S3).

**Figure 4:**
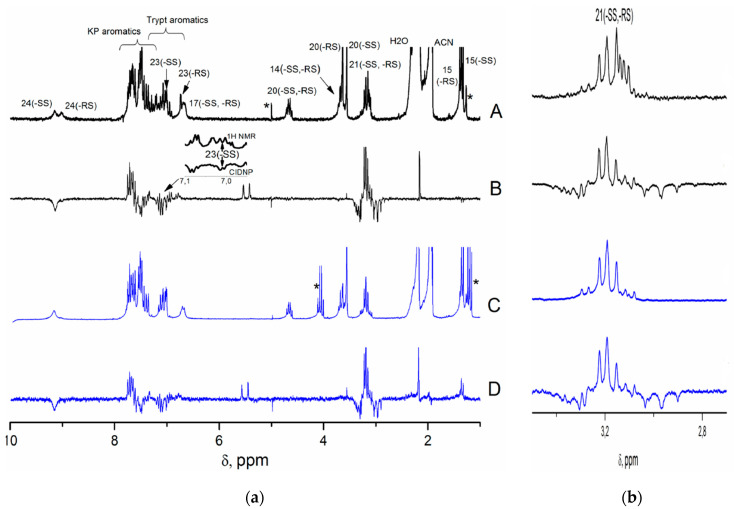
(**a**) ^1^H NMR and CIDNP spectra of racemic mixture (*S/R*)-KP-(*S*)-Trp (A,B) and (*S*)-KP-(*S*)-Trp (C,D) in acetonitrile-d_3_, (**b**) expanded region for CH_2_ protons. Asterisks denote image artifacts and synthesis and solvent-related impurities.

Indeed, NMR spectra of the (*S/R*)-KP-(*S*)-Trp dyad recorded under UV irradiation demonstrate the CIDNP effects for protons of the initial dyads (Figure 4). For (*S,S*)-diastereomer, the aromatic protons of KP and indole, as well as indole NH and methylene protons of the Trp fragment are polarized. At the same time, for the (*R,S*) analogue, the hyperpolarization of methylene and indole protons is much weaker than those detected for the (*S,S*)-diastereomer (Figure 4).

This CIDNP pattern, namely, the appearance of hyperpolarization at protons of initial dyad, indicates reversible ET. As can be seen from Table 1, intramolecular ET between KP (acceptor) and Trp (donor) could occur in several ways: from Trp in excited state (S_1_) to KP in ground state (S_0_) (path 1), and from Trp in ground state (S_0_) to KP in excited states (path 2). The comparison of absorption spectra of components leads to the conclusion that under the laser irradiation (308 nm) of dyads, light is absorbed by both chromophores (Figure S6).

CIDNP analysis, carried out in accordance with the rules for high magnetic fields, shows that observed effects correspond to the case when biradical zwitterion is formed from the singlet excited state of chromophore (path 1) [14]. Details of CIDNP analysis are presented in Table S1.

To solve the question of which chromophore in a singlet excited state is involved in CIDNP formation, the CIDNP effects in photoinduced interaction of KP and N-acetyltryptophan methyl ether in solution were studied. CIDNP pattern turned out to be dependent on the ratio of reagents: at an equimolar ratio, the CIDNP signs were the same as during the photolysis of dyad. In the case of KP excess in solution, the signs were opposite and conformed to radical ion pairs with triplet precursor, i.e., KP, like other ketones, reacted from a triplet excited state (Figure S5). In summary, these results show that the prevailing mechanism of ET in (*S/R*)-KP-(*S*)-Trp dyad is the quenching of Trp in S_1_ state by KP in ground state (S_0_).

**Figure 5:**
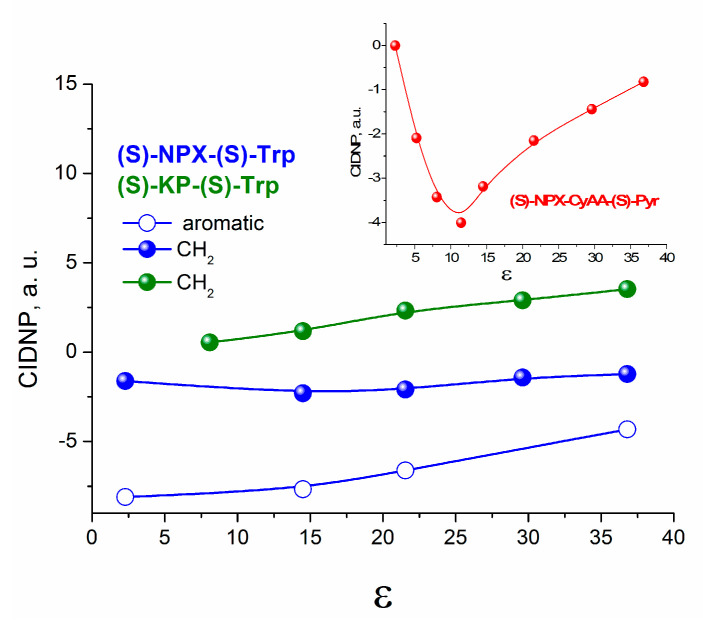
CIDNP dependence on solvent dielectric constants for (*S*)-KP-(*S*)-Trp dyad (green) in comparison with earlier studied systems containing (*S*)-NPX-(*S*)-Trp (blue) and (*S*)-NPX-(*S*)-N-methylpyrrolidine (red).

Concerning the exciplex formation, the weak dependence of the CIDNP efficiency on polarity does not testify in favor of its formation (Figure 5). In the presence of exciplex that is in rapid equilibrium with the biradical zwitterion, a more pronounced dependence of CIDNP on polarity is usually observed [6] (Figure 5).

The curves in Figure 5 represent the dependences of CIDNP effectivity of different protons of (*S*)-KP-(*S*)-Trp and ‘naproxen-tryptophan’ ((*S*)-NPX-(*S*)-Trp) dyads [6] on the dielectric constant. No experimental evidence was found for exciplex formation for the last dyad. Final conclusions about the formation of the exciplex under the UV irradiation of (*S/R*)-KP-(*S*)-Trp will be made below on the basis of time-resolved fluorescence studies.

**Figure 6:**
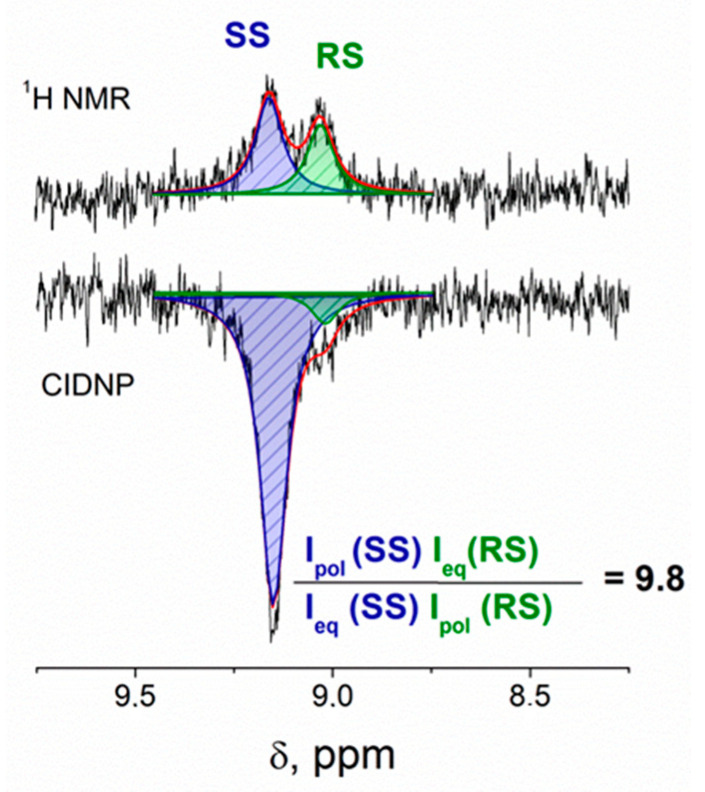
The comparison of NH protons net CIDNP intensities of diastereomers.

In accordance with the assumption that the fractions of the (*S,S*)- and (*R,S*)-diastereomers quenched through ET have to be proportional to their hyperpolarization (Figure 6), we will try to estimate the ET contributions using CIDNP enhancement coefficients. For that, it is necessary to clarify what the difference of CIDNP enhancement coefficients depends on.

As indicated in work [12], the CIDNP enhancement coefficients ratio of (*S,S*) and (*R,S*)-diastereomers (K) is defined as:

(3) $$K=\frac{I_{\mathrm{pol}}^{\mathrm{SS}}\times I_{\mathrm{eq}}^{\mathrm{RS}}\times {\left[\mathrm{BZ}\right]}_{\mathrm{RS}}}{I_{\mathrm{eq}}^{\mathrm{SS}}\times I_{\mathrm{pol}}^{\mathrm{RS}}\times {\left[\mathrm{BZ}\right]}_{\mathrm{SS}}},$$

where I_pol_—integral intensity of polarized signals of some protons; I_eq_—equilibrium signal of the same protons, [BZ]—concentration of biradical zwitterion. Here, K is the ratio of the absolute enhancement coefficients of CIDNP for diastereomers. It should be emphasized that I_pol_ values are determined by the magneto resonance parameters of paramagnetic precursors of (*R,S*)- and (*S,S*)-diastereomers: the values of the HFI constants, the differences in the g-factors and the lifetimes of the biradical zwitterions [12,14].

As mentioned above, hyperpolarization in chiral linked systems demonstrates spin selectivity, which allows us to expect that CIDNP enhancement coefficients might be different for (*R,S*)- and (*S,S*)-diastereomers [12]. In the case of previously studied diastereomers of (*S/R*)-NPX-(*S*)-Trp and (*S/R*)-NPX-(*S*)-N-methylpyrrolidine dyads, the K values were about two. According to [12,13], this was the result of two-fold difference in the hyperfine coupling constants (HFI) of aromatic protons of NPX and the methyl protons of N-methylpyrrolidine fragment in biradical zwitterions in (*R,S*) and (*S,S*) configurations. On the other hand, the proton hyperpolarization of diastereomers of products formed from (*S/R*)-KP-(*S*)-N-methylpyrrolidine dyad differ less: the CIDNP of products from (*S,S*)-diasteromer is only 30% higher than for the (*R,S*) analogue [17]. The difference in spin selectivity in the dyads with naproxen and ketoprofen may be due to differences in the efficiency of back ET in the biradical zwitterions of these dyads. These can be both differences in lifetimes and in the biradical zwitterion configuration [12,13]. The above considerations, as well as the fact that the effective HFI constants of the methylene protons of the tryptophan and methyl protons of the pyrrolidine fragments are of the same order of magnitude, allow one to conclude that it is reasonable to use the data on the spin selectivity of the dyad with ketoprofen and N-methylpyrrolidine.

If we assume that the difference of 30% is characteristic for the CIDNP coefficients for (*S*)-KP-(*S*)-Trp and (*R*)-KP-(*S*)-Trp diastereomers, then the ratio of biradical zwitterions concentrations can be roughly estimated as 9.8/1.3 = 7.5.

Thus, if CIDNP is determined by the ET contribution to the quenching of dyad fluorescence, then the ratio of these contributions for (*S,S*) and (*R,S*) will be proportional to the ratio of biradical zwitterions concentration of diastereomers: about 7.5.

### 2.2. Fluorecsence Quenching

**Figure 7:**
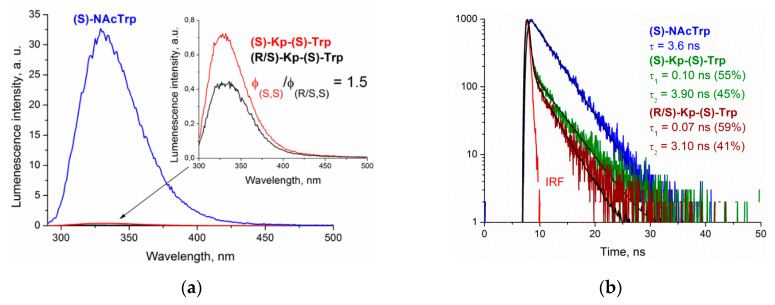
(**a**) Fluorescence spectra of isoabsorptive solutions of (*S*)-NAcTrp (blue), (*R/S*)-KP-(*S*)-Trp (black) and (*S*)-KP-(*S*)-Trp (red) in acetonitrile (λ_ex_ = 280 nm) in 1 cm cuvette. Concentrations were on the order of 10^−4^ M. (**b**) Fluorescence decay traces of (*S*)-NAcTrp (blue), (*S*)-KP-(*S*)-Trp (green) and (*R/S*)-KP-(*S*)-Trp (wine) in acetonitrile at 330 nm (λ_ex_ = 300 nm). IRF—instrument response function.

The emission spectra of the (*S,S*) dyad and the racemic mixture of (*R,S*) and (*S,S*) in comparison with (*S*)-N-acetyltryptophan ((*S*)-NAcTrp) in acetonitrile are shown in Figure 7. The fluorescence spectra of the dyads have the typical emission band of Trp chromophore with a maximum at 330 nm. Note that exciplex formation under UV irradiation was not found in (*S/R*)-KP-(*S*)-Trp (Figure 7).

The comparison of the fluorescence quantum yields of the (*S*)-NAcTrp and dyad showed a significant decrease of these values for the dyad: 97.5% of Trp fluorescence in the (*S,S*)-dyad was quenched. The ratio of fluorescence quantum yields of the (*S,S*)-dyad and the (*S,S*), (*R,S*) mixture was 1.5. Taking into account that the racemic mixture contains 47% (*R,S*) and 53% (*S,S*), the ratio of fluorescence quantum yields (*S,S*)/(*R,S*), with a value of 5.4.

**Figure 8:**
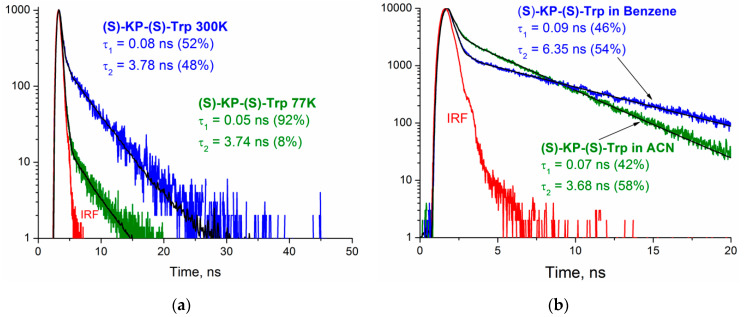
(**a**) Fluorescence decay traces of (*S*)-KP-(*S*)-Trp in acetonitrile at 77 (green) and 300 K (blue) and λ_em_ = 330 nm (λ_ex_ = 270 nm). (**b**) Fluorescence decay traces of (*S*)-KP-(*S*)-Trp in acetonitrile (green) and benzene (blue) at 330 nm (λ_ex_ = 270 nm); IRF—instrument response function. The corresponding fluorescence spectra are shown in Figure S7.

The fluorescence decay traces were fitted in two exponential models, similar to those in Trp of proteins [22,26]. The manifestation of two-exponential decay is usually associated with the presence of several conformers for Trp with the distribution of dependences on the solvent and temperature [20,24,26,29,30]. Figure 8 clearly shows the change in the kinetic traces of the (*S*)-KP-(*S*)-Trp dyad at different temperatures and solvent polarities. This confirms that the contributions from individual conformations of dyads resulted in complex decay. According to the reference data, different conformations in solutions are characteristic not only of Trp, but also for KP [31].

Therefore, the observations of extremely low fluorescence quantum yields of Trp in dyads (0.004 and 0.0007) and the variation of lifetime with the change of solvent and temperature are consistent with the reference data for different proteins, where similar behavior of Trp is considered to be a well-known fact [24,26,29,30], wherein the reason for small outputs of Trp fluorescence in proteins is believed to be quenching via ET and RET [24,26]. In this case, ET is supposed to be the result of charge transfer from the indole of the Trp fragment to the peptide bonds (NH-C=O), while RET occurs between adjacent amino acid residues [24,26].

Note that for some dyads the fluorescence from the Trp moiety was not detected at all, for example, under the UV irradiation of the NPX-Trp dyad [23]. In the case of the ‘flurbiprofen–tryptophan’ (FBP-Trp) dyad, quantum yields of Trp fluorescence were also appreciably low [22].

As follows from Table 1, the energy characteristics of the (*S/R*)-KP-(*S*)-Trp dyad partners point to the possibility of several ways of Trp fluorescence quenching: in addition to ET, energy transfers between Trp and KP according to Dexter mechanism and the RET from Trp in S_1_ to KP in S_0_ state are also allowed [22,25]. Since the present article focuses on the investigation of processes involving the singlet excited state of Trp by fluorescence and CIDNP techniques, energy transfer by the Dexter mechanism is beyond the scope of this consideration.

**Figure 9:**
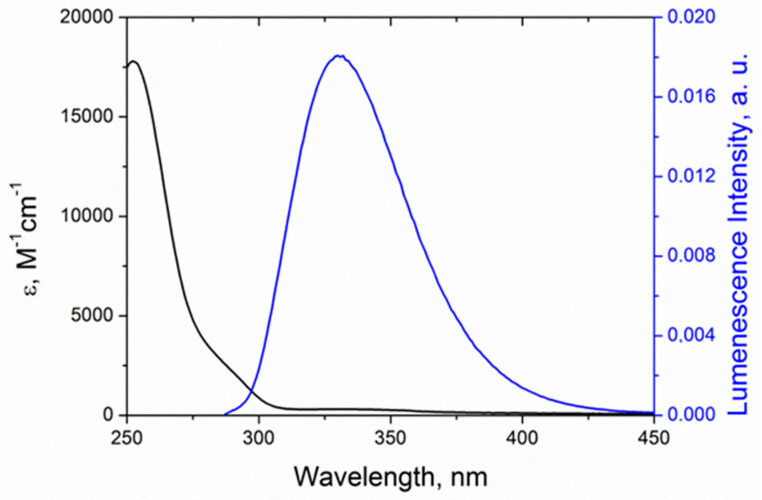
Emission spectrum of Trp donor (blue) and absorption spectrum of KP acceptor (black) in acetonitrile.

The confirmation of RET feasibility is the spectral overlap of Trp emission (maximum at 330 nm) and absorption of KP (low intensity shoulder with maximum at 350 nm) (Figure 9).

To evaluate the contribution of the RET mechanism, one should consider the residual tryptophan fluorescence. Since the fluorescence spectra of Trp moiety in the (*S/R*)-KP-(*S*)-Trp dyad are close to the individual Trp emission spectrum, it is reasonable to assume that values of non-radiative, radiative and intersystem crossing constants are kept. Therefore, the average quenching rate constant *k_Q_* was determined by

(4) $$\frac{\varphi_{0}}{\varphi }=1+k_{Q}\tau_{0},$$

where *φ*_0_ and *τ*_0_—fluorescence quantum yield and lifetime of individual NAcTrp (0.16 and 3.6 ns), *φ*—fluorescence quantum yield of the dyad (0.004 for (*S,S*) and 0.0007 for (*R,S*)). In addition, the ratio of quenching rate constants of diastereomers is

(5) $$\frac{k_{Q}^{RS}}{k_{Q}^{SS}}=\frac{\left(\varphi_{0}-\varphi^{RS}\right)\varphi^{SS}}{\varphi^{RS}\left(\varphi_{0}-\varphi^{SS}\right)},$$

where superscripts denote quantum yields and quenching constants of (*S,S*)- and (*R,S*)-diastereomers, accordingly.

Thus, the ratio of the quenching rate constants for the (*R,S*) and (*S,S*) configurations, determined based on (5), is 5.8. At the same time, the concentration of biradical zwitterions for (*S,S*)-diastereomer is about 7.5 times higher than in the case of (*R,S*). The comparison of these values allows one to conclude that (*R,S*)-diastereomer of a dyad is quenched mainly through the RET mechanism, while ET is essential for the (*S,S*) configuration. There are other examples in the literature of the stereoselectivity of Trp fluorescence quenching associated with RET [22,26,32]. For instance, the stereoselective Trp fluorescence quenching in FBP-Trp dyad through RET has been described in [32] using quantum chemical calculation and molecular modeling. The authors found that the stereoselectivity in this dyad arises from an almost orthogonal arrangement of the transition dipole moments of flurbiprofen and Trp in the (*S,S*)-diastereomers that explain the 2-3 times slower dynamic quenching [32].

**Figure 10:**
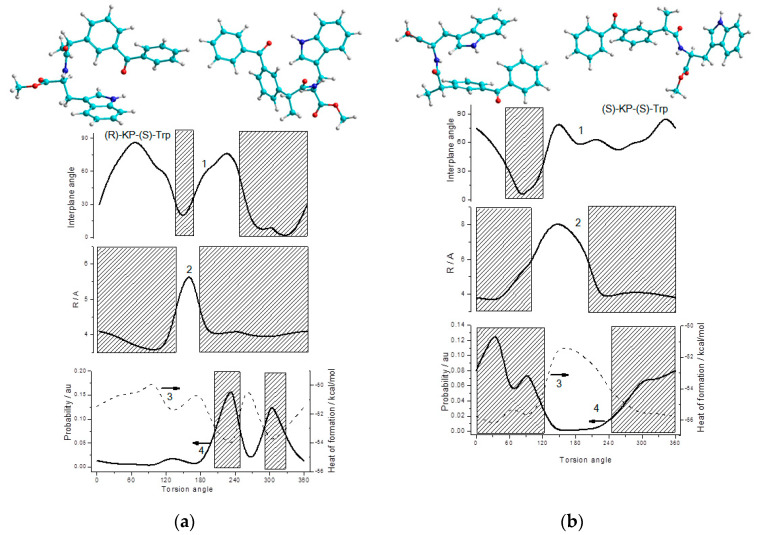
The dependences of: interplane angle (1), intercenter distance (2), arbitrary probability of configuration (3), heat of formation (4) on the torsion angle values calculated for (**a**) (*R*)-KP-(*S*)-Trp, (**b**) (*S*)-KP-(*S*)-Trp dyads. Filled rectangles mark the mostly probable ranges and the ranges with the favorable conditions for ET. Calculations were performed by semi-empirical AM1 method using Hyperchem 8 software.

We also made an attempt to trace the relationship between the heats of formation, angles and distances between partners at different dihedral angles of rotation of the bridging carbon atoms for the diastereomers of (*S/R*)-KP-(*S*)-Trp dyad (Figure 10, Figures S8–S11). Analysis of the dependences of the heat of formation of diastereomers on the torsion angle around the C-C bond of the bridge showed no significant differences between (*R,S*)- and (*S,S*)-diastereomers distances (Figure 10). It turned out that the most stable conformations with minimal free energies for (*S,S*)- and (*R,S*)-diastereomers of the dyad are characterized by the very close minimal distances between carbonyl carbon of KP and nitrogen of indole ring (r_SS_ and r_RS_ ~ 4.0 Å). However, it should be noted that (*S,S*)-diastereomer has a wider region where, at minimum energies, large distances between partners are observed (~6.0 Å, Figure 10b).

In addition, the difference of distances between partners in (*S,S*)- and (*R,S*)-diastereomers was confirmed by 1D NOE experiment (Figure S4). Therefore, during radio frequency irradiation of the proton located at the 23 position of the indole ring of (*R*)-KP-(*S*)-Trp, a cross peak is observed at the ortho-/para-protons of KP, while upon selective excitation of this proton in (*S*)-KP-(*S*)-Trp this signal is not observed. As a rule, positive NOE cross peaks arise when interproton distance is no greater than 4.5 Å. This indicates that Trp and KP rings in (*S,S*)-diastereomer are in fact farther apart from each other than 4.5 Å.

According to Förster theory [26,32], another important attribute that determines the efficiency of RET is the relative orientation in space of the transition dipoles of the donor and acceptor. Dipole−dipole orientation factor κ depends on the angles between these dipoles and the vector joining the donor and the acceptor and the angle between the planes in which dipoles are located.

We assume that in our dyad, the dipoles are localized in the planes of the indole ring and the C-C(O)-C group of KP, and the transition of KP to the excited state does not greatly change its direction. Taking this assumption into account, differences in the calculated dependences of the interplanar angles between the donor and the acceptor in the (*R,S*)- and (*S,S*)-diastereomers from the dihedral angles can be used to interpret the difference in the efficiency of RET and ET for (*R,S*) and (*S,S*).

Judging by the calculated dependences shown in Figure 10, both (*R,S*)- and (*S,S*)-diastereomers have regions where structures with minimum energy also demonstrate a mutual arrangement of the indole and KP aromatic rings close to the plane (Figure 10). However, the (*R,S*)-diastereomer demonstrates a deeper well for structures with a flat arrangement of partners providing effective RET (Figure 10a). Thus, this pattern does not contradict the experimental result, where more efficient fluorescence quenching through RET was observed for the (*R,S*)-diastereomer.

On the other hand, for the (*S,S*)-diastereomer, comparable areas with the minimum energy corresponding to arrangement with large interplane angles, unfavorable for RET, are observed (Figure 10b). The presence of such “expanded” structures allows one to suggest that (*S,S*)-diastereomer would more easily undergo the conformational rearrangement necessary for the formation of biradical zwitterions.

**Figure 11:**
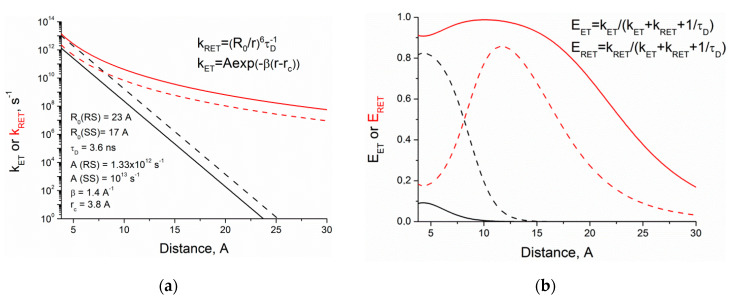
Dependence on distance of (**a**) the rate constants for RET (red curve) and ET (black); (**b**) the calculated efficiencies for RET (red) an ET (black). The curves for (*R,S*)- and (*S,S*) diastereomers are indicated by solid and dashed lines, respectively.

To trace the efficiencies of RET and ET mechanisms of fluorescence quenching of (*S,S*)- and (*R,S*)-diastereomers depending on the distance between partners, simulation of the k_RET_ and k_ET_ was carried out using known expressions for evaluation of quenching constants [26,27,28]. A qualitative picture of the dependence of the rate constant on distance for RET and ET (obtained using the simplest Marcus expression) is shown in Figure 11.

Förster radius (*R*_0_) was estimated from the spectral properties of the donor and the acceptor (Figure 9) and the donor quantum yield (*φ_D_* = 0.16) by the following equation [26]:

(6) $$R_{0}=0.211{\left(\kappa^{2}n^{-4}\varphi_{D}J\left(\lambda \right)\right)}^{1/6},$$

where *κ*^2^ is the factor describing donor–acceptor orientation taken under the assumption that these orientations do not change during the lifetime of excited state (0.476), *n*—refractive index of the medium (1.344), *J*(*λ*)—overlap integral between the donor emission and the acceptor absorption (7.22 × 1013 nm^4^·M^−1^·cm^−1^). The value of *β* for various aromatic systems is about 1–2 Å^−1^ [28]. The differences in RET and ET rate constants for diastereomers were taken from experimental data. The ratio of diastereomers ET rate constants as shown above is 7.5, and this difference was attributed to the pre-exponential factor. The value of RET rate constant in (*R,S*)-diastereomer was assumed to be six times higher than for (*S,S*)-. This, in turn, implies differences of 1.35 times in Förster radii that can account for the different orientation factor κ of diastereomers [32].

Comparison of the *k_RET_* and *k_ET_* dependences on the distance between donor and acceptor shows the difference of diastereomers’ RET and ET rate constants (Figure 11a). Figure 11b presents the dependence of the diastereomers quenching efficiency through both mechanisms on the distance between partners. The distances between partners, characteristic for each diastereomer, are r_SS_~4.0 Å, r_RS_~6 Å. According to the calculation results, the dependences of RET and ET efficiencies on distance, as shown in Figure 11b, are remarkably different. Comparable with the experimental data, differences in diastereomer quenching efficiency obtained at small distances (8 times for ET and 4.5 for RET) indicate the adequacy of the parameters used.

In conclusion, it should be mentioned that the use of the dyad model system made it possible to detect quenching of Trp fluorescence via ET, which had hitherto only been assumed in several studies [20,22,24,29]. The advantage of the dyad model systems is that partners linked by a strong donor–acceptor interaction demonstrate that full ET resulted in the formation of radical ions that can be detected by physical methods. However, even in such systems, as shown above, RET is more effective. The latter allows us to express doubt that ET involving a peptide bond can indeed compete with RET in the proteins of living systems, as suggested in [18,20,29].

Actually, under UV irradiation of chiral linked systems, ET was proved to occur between the two partners, in the dyads under discussion these are the donor—the Trp moiety—and the acceptors—naproxen, flurbiprofen and ketoprofen residues [22,23]. Note that, despite the fact that all of the mentioned dyads include an NН-С=О fragment, no signs of charge transfer between this group and Trp residues were observed either in the ground or in the excited state of dyads, neither by the CIDNP nor by absorption spectra [24,26].

It is also worth noting that in all previously studied chiral dyads, the fluorescence of (*R,S*)-diastereomers was quenched more efficiently than for (*S,S*) analogues [5,6,7]. This also occurs in single-chromophore systems, where fluorescence quenching is exclusively the result of ET [4,5,6,7,12]. Additionally, the results of this study show that one of the most defining properties of chiral systems is the stereospecificity of their activity.

## 3. Materials and Methods

### 3.1. Synthesis of the (S/R)-Ketoprofen-(S)-Tryptophan Dyad

The synthesis was carried out according to the scheme described in detail in the Supplementary Materials.

### 3.2. Spectroscopic Measurements

All UV spectroscopic measurements were performed using quartz cuvettes of 1 cm and 1 mm optical length. Acetonitrile (Kriochrome, Saint-Petersburg, Russia) and benzene (PanReac, Barcelona, Spain) were used as solvents. Spectra and kinetic curves of luminescence were recorded with an Edinburgh Instruments FLSP-920 spectrofluorimeter with either a Xenon lamp or laser diodes EPLED-300 and EPLED-270 (λ_ex_ = 300 and 270 nm, pulse duration 0.6 ns) as excitation sources. The kinetic traces were fitted by exponential decay functions using a reconvolution procedure. Processing of kinetic curves (programs of Edinburg Instruments—DATA PROCESSING and FAST) together with IRF due to mathematical convolution allowing the determination of the times of photophysical processes with a resolution of about 100 ps. The absorption spectra were recorded using an Agilent 8453 spectrophotometer.

### 3.3. NMR Measurements

^1^H NMR spectra were obtained on a Bruker Avance HD III NMR spectrometer (500 MHz ^1^H operating frequency, τ(π/2) = 10 μs). CIDNP experiments were performed on a Bruker DPX-200 NMR spectrometer (200 MHz ^1^H operating frequency, τ(π/2) = 2.5 μs). A Lambda Physik EMG 101 MSC eximer laser was used as a light source (308 nm, 100 mJ at output window, 20 mJ/pulse in sample volume, pulse duration 15 ns) in the CIDNP experiments. The samples in standard 5 mm Pyrex NMR tubes were irradiated directly in the NMR probe of Bruker DPX200 NMR spectrometer. The samples were bubbled with argon for 10-15 min to remove dissolved oxygen just before photolysis. Acetonitrile-d_3_ (Aldrich, D 99.8%, St. Louis, USA) and benzene-d_6_ (Aldrich, D 99.6%, St. Louis, USA) were used as solvents.

Pseudo steady-state photo-CIDNP (PSS).

The PSS experiments were performed using a Bruker DPX-200 NMR spectrometer and a standard pulse sequence: presaturation–delay 1–pulse τ(π)–delay2 (16 laser flashes with repetition rate 50 Hz during delay2)–observation pulse τ(π/2)–acquisition. Delay1/delay2 ≈ 1.1 to remove residual signals of solvents and solutes. After laser irradiation, the ^1^H spectra of products were recorded on Bruker Avance HD III NMR spectrometer.

## 4. Conclusions

The main results of this work are the demonstration of the possibility of independent observation of two mechanisms of tryptophan fluorescence quenching in the (*S*)-KP-(*S*)-Trp and (*R*)-KP-(*S*)-Trp dyads’ diastereomers. Therefore, the joint analysis of CIDNP and fluorescence data shows that the singlet excited state dyad is quenched through the RET and the ET mechanisms. The ratio of observed quenching rate constants of (*R,S*)- and (*S,S*)-diastereomers indicates that the main quenching channel of both diastereomers is RET. At the same time, CIDNP observation indicates a manifestation of the ET mechanism, which is more effective for the (*S,S*)-diastereomer. Therefore, the different contribution of the two mechanisms to the quenching of (*R,S*) and (*S,S*) excited states is evidence of stereoselectivity of both ET and RET.

## Figures and Tables

**Table 1:** Energy of singlet and triplet excited states (*E^S^*, *E^T^*) and oxidative (*E_ox_*) and reduction (*E_red_*) polarographic half-wave potentials of tryptophan and ketoprofen molecules.

Compound	*E^S^* (eV)	*E^T^* (eV)	*E_ox_* (V)	*E_red_* (V)
Tryptophan	4.17 [22]	3.10 [22]	1.02 [22]	-
Ketoprofen	<4.0	3.15 [25]	-	1.24 [25]

## Supplementary Materials

Supplementary materials can be found at https://www.mdpi.com/1422-0067/21/15/5370/s1.

## Abbreviations

CIDNP	Chemically induced dynamic nuclear polarization
ET	Electron transfer
RET	Resonance energy transfer
NSAIDs	Non-steroidal anti-inflammatory drug
