# Supplementary material for: Stereoselectivity of Electron and Energy Transfer in the Quenching of (S/R)-Ketoprofen-(S)-Tryptophan Dyad Excited State
Source: Int J Mol Sci. 2020 Jul 28;21(15):5370. doi: 10.3390/ijms21155370 (PMC7432585; doi:10.3390/ijms21155370)
Supplement: Supplementary file 1 [file ijms-21-05370-s001.pdf]

Stereoselectivity of Electron and Energy Transfer in the Quenching of (S/R)-Ketoprofen-(S)-Tryptophan Dyad Excited State

Aleksandra A. Ageeva [1*], Simon V. Babenko [1], Ilya M. Magin [1], Victor F. Plyusnin [1,2], Polina S. Kuznetsova [1,2], Alexander A. Stepanov [1], Sergey F. Vasilevsky [1], Nikolay E. Polyakov [1], Alexander B. Doktorov [1], Tatyana V. Leshina [1]

[1] Voevodsky Institute of Chemical Kinetics and Combustion SB RAS, 630090 Novosibirsk, Russia; simonb683@gmail.com (S.V.B.); magin@kinetics.nsc.ru (I.M.M.); plyusnin@kinetics.nsc.ru (V.F.P.); Pol0596@yandex.ru (P.S.K.); stepanov@kinetics.nsc.ru (A.A.S.); vasilev@kinetics.nsc.ru (S.F.V.); polyakov@kinetics.nsc.ru (N.E.P.); doktorov@kinetics.nsc.ru (A.B.D); leshina@ngs.ru (T.V.L.)

[2] Novosibirsk State University, Novosibirsk, Russia

* Correspondence: al.ageeva@gmail.com; Tel.: + 7-383-3332947 (N.E.P)

Table of contents

1. Synthesis of (S/R)-ketoprofen-(S)-tryptophan dyad
2. NMR spectra of (S/R)-ketoprofen-(S)-tryptophan dyad
3. CIDNP analysis
4. Absorption and fluorescence spectra of the dyad
5. Quantum chemical calculations of possible configurations of diastereomers of a dyad (S/R)-ketoprofen-(S)-tryptophan

1. Synthesis of (S/R)-ketoprofen-(S)-tryptophan dyad

The synthesis was carried out from ketoprofen and tryptophan according to the following scheme:

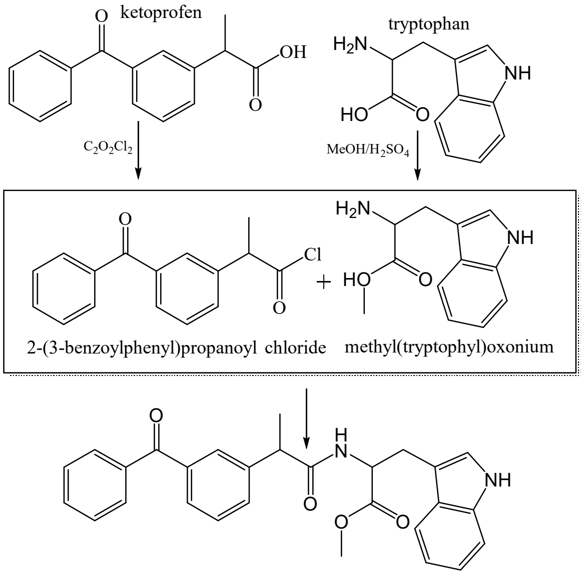

Scheme 1. Synthesis of (R/S)-KP-(S)-Trp dyad.

Materials. All reagents were ACS grade, and were used without further purification. The UV-254 plates were used for TLC analysis. The IR-spectra were recorded in KBr pellets. Mass spectra (HRMS) were measured on at 70 eV. NMR spectra were recorded at 500 (^{1}H) at 25 °C. Chemical shifts (*δ*) are given in ppm with reference to the residual signals of [D_1] chloroform (1H: *δ* = 7.24 ppm). The mass spectra (High Resolution GC/MS) were measured by the direct injection method (the temperature of the ionization chamber was 220−270 °C and the ionization voltage was 70 eV).

Solution of methyl tryptophanate (0.22 g, 1 mmol) in 3 mL toluene and 0.3 mL Et3N (2.1 mmol) was added within 5 minutes to solution of 2-(5-benzoylthien-2-yl)propanoyl chloride (ketoprofen chloride) (0.27 g, 1 mmol) in 1 mL of toluene at room temperature [1,2]. The reaction mixture was stirred at room temperature for 18 h. The solvent was removed *in vacuo.* The precipitate was washed with ethyl oxide. The solvent was removed *in vacuo* and the residue was purified by column chromatography (ethyl acetate/hexane). Then solvents were evaporated to dryness under reduced pressure. Yield: 0.23 g, 51%, mp 53-55 °C. ^{1}H NMR (500 MHz, $CDCl_3$) δ 1.53-1.54 (m, 3H); 3.28-3.30 (m, 2H); 3.60-3.61 (m, 1H); 3.67 (s, 3H); 4.84-4.88 (m, 1H); 5.85-5.87 (m, 1H); 6.75-6.75 (m, 1H); 7.09-7.12 (m, 1H); 7.17-7.20 (m, 1H); 7.34-7.35 (m, 1H); 7.40-7.43

(m, 1H); 7.47-7.50 (m, 4H); 7.60-7.63 (m, 2H); 7.6-7.68 (m, 1H); 7.77-7.79 (m, 2H); 8.14 (br.s, 1H). HRMS, ^{1}H NMR (125 MHz, $CDCl_3$) 17.9; 27.0; 46.7; 52.2; 52.5; 109.4; 111.2; 118.2; 119.5; 122.1; 122.6; 127.2; 128.2; 128.7; 128.8; 128.9; 130.0; 131.5; 132.5; 135.8; 137.0; 137.9; 140.8; 171.9; 172.9; 196.7. δ Found: m/z 454,1886 [M]+. $C_{28}H_{26}N_2O_4$. Calcd.: M=454,1887. IR, cm^{-1}, ν: 1649 (C=O); 1735 (C=O).

The analysis of the dyads' purity was carried out by thin layer chromatography (TLC), IR, high resolution GC / MS and NMR techniques. Dyads' purity studies did not reveal the presence of Trp impurities. The possibility of its formation during the photodecomposition of the dyad was excluded by monitoring this process by high resolution NMR.

2. *NMR spectra of (S/R)-ketoprofen-(S)-tryptophan dyad*

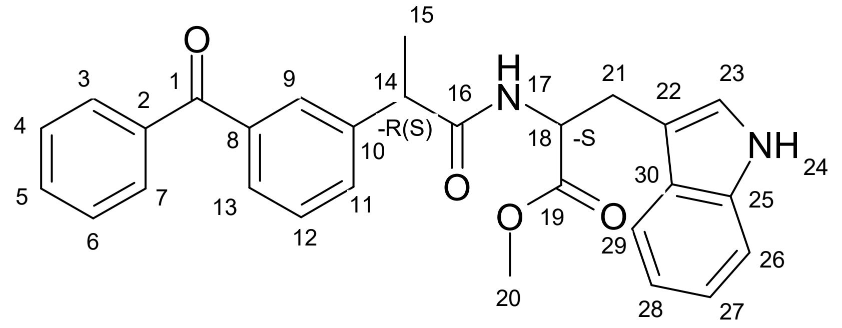

Figure S1. (S/R)-ketoprofen–(S)-tryptophan dyad

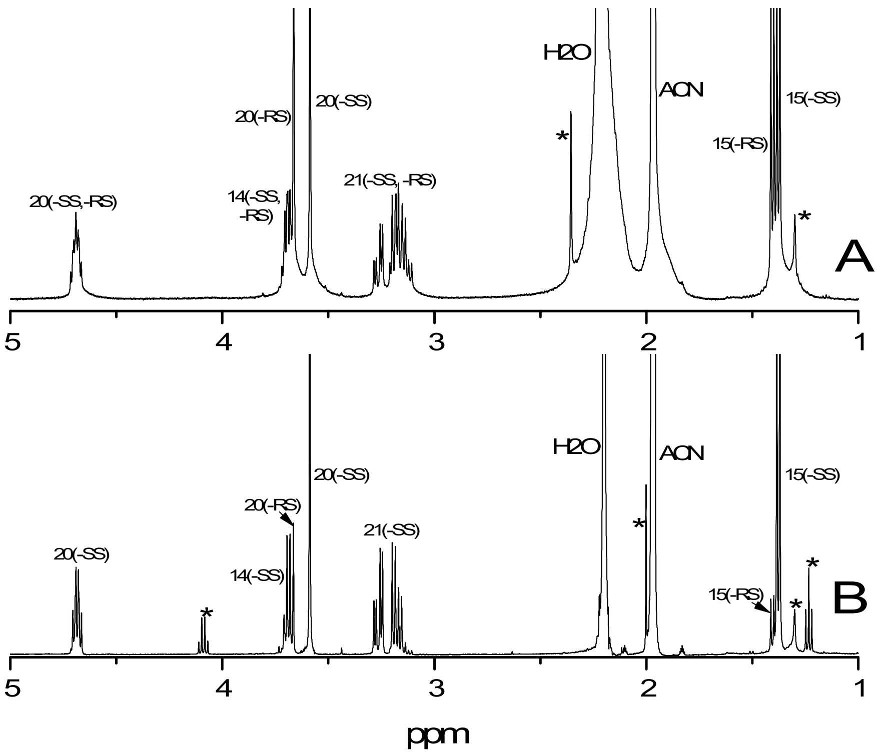

Figure S2. The aliphatic part of ¹H NMR spectra (1-5 ppm) of racemic (S/R)-KP-(S)-Trp (A) and (S)-KP-(S)-Trp (B) in ACN-d_3. Asterisks denote synthesis- and solvent-related impurities like ethyl acetate. The (S,S)-isomer contains admixture of (R,S)-isomer traceable in the spectrum.

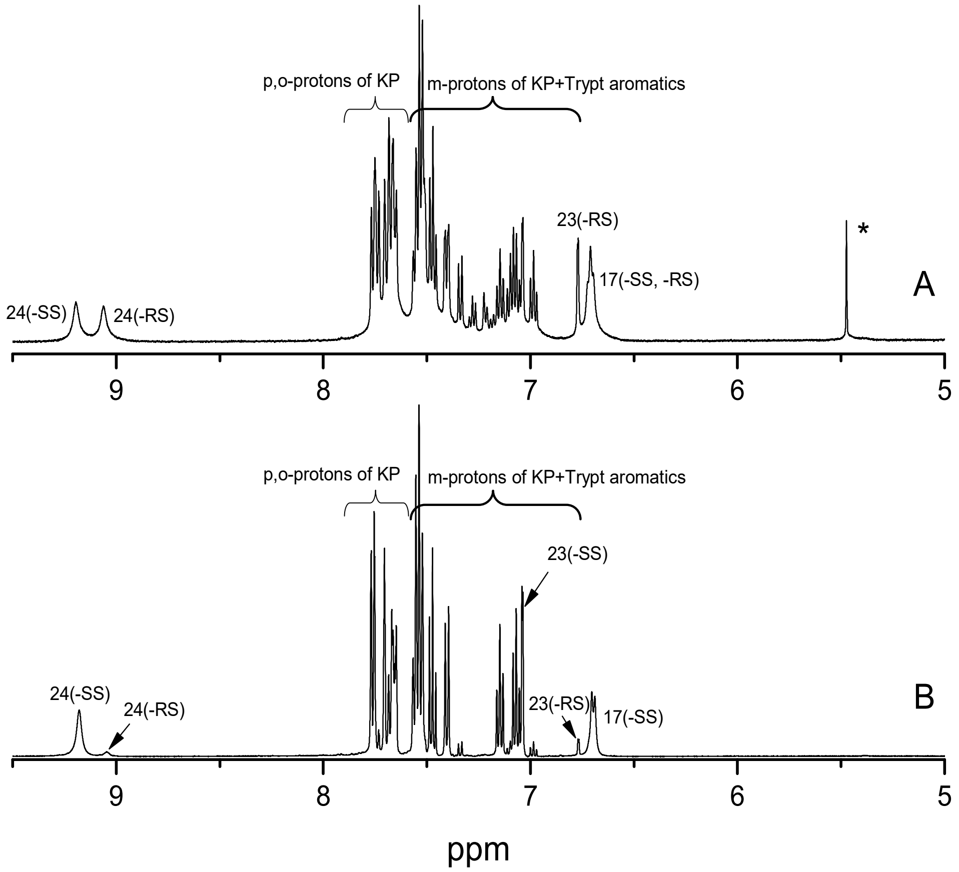

Figure S3. The aromatic part (5-9.5 ppm) of ^{1}H NMR spectra of racemic (S/R)-KP-(S)-Trp (A) and (S)-KP-(S)-Trp (B) in ACN-d3. Asterisks denote synthesis- and solvent-related impurities.

^{1}H NMR spectra of racemic (S/R)-KP-(S)-Trp and 1D NOE polarization transfer are presented below. Protons located at 23 position of the indole ring of tryptophan fragment (Figure S1) of the both isomers of dyad (since they are strongly spaced in the spectra (Figures S2-S3)) were exposed to RF irradiation. These are intense emission lines in the spectra. All absorption lines are those protons that are located next to the irradiated protons (as a rule, protons located at a distance of up to 4.5 Å are visible). The number of scans and concentration are the same. When 23 proton of (R,S) dyad is irradiated, a cross peak is observed at the ortho/para protons of KP (marked with an oval), while when the same proton of (S,S) dyad is excited, there is no such peak. It let us to suggest that tryptophan and ketoprofen rings in (R,S) dyad are closer to each other than in (S,S) analog.

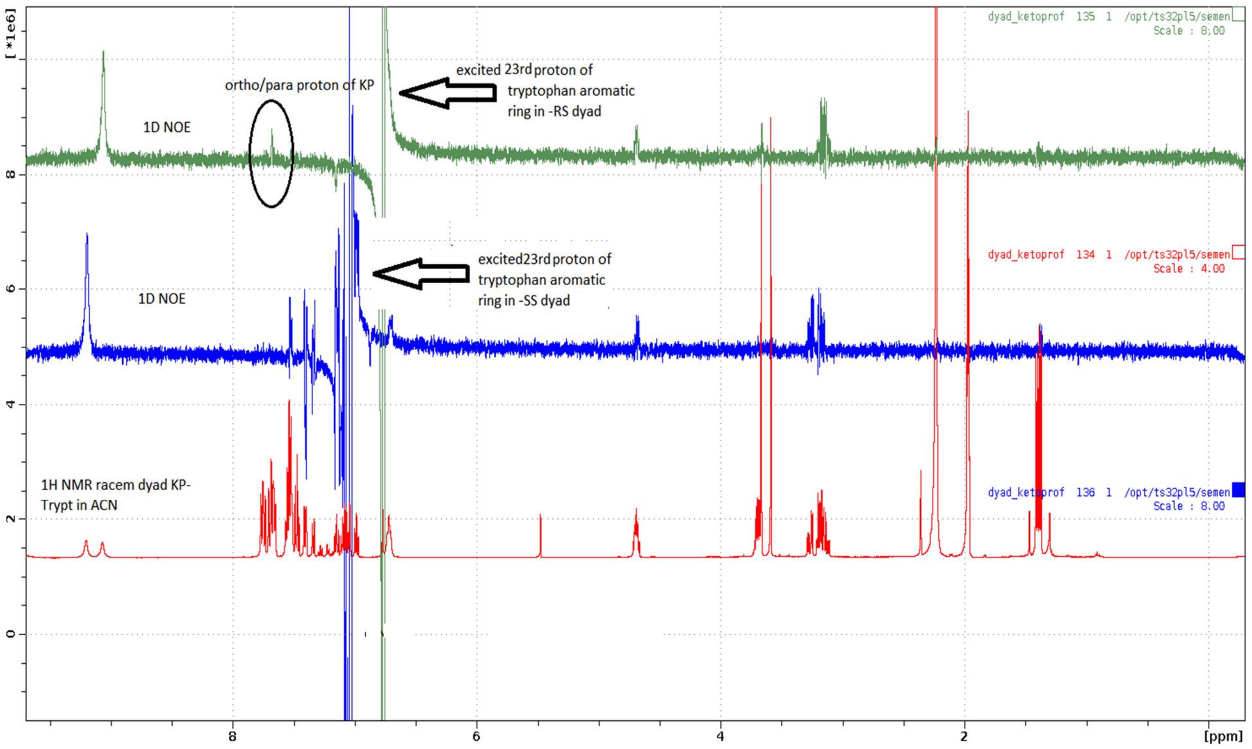

Figure S4. 1D NOE spectra of (R,S) (green) and (S,S) (blue) diastereomers. When 23 proton of tryptophan fragment of (R,S) dyad is excited, a cross peak is observed at the ortho/para protons of KP (marked with an oval), while when this proton of (S,S) dyad is excited, there is no such peak.

3. *CIDNP analysis*

An analysis of CIDNP spectra detected at high magnetic field can be obtained by well-known Kaptein's rules, modified by Closs [3], taking into account the possibility of biradical-zwitterion recombination from both spin states. For the net effect, the sign can be determined by the following parameters

$$\Gamma_i = (\mu)(\varepsilon)(\Delta g)(a_i)(\gamma),$$

where μ denotes the initial spin multiplicity (+ for triplet, − for singlet), ε the type of reaction leading to the observed products (+ for cage product, − for escaped products), Δg = g_1−g_2 the sign of difference in g-factors of the two radicals (g_1 is the g-factor of the radical ion with nucleus under observation), a_i the sign of the HFI constant and 'exit channel' factor γ (+ for singlet exit channel, – for triplet). The sign product of these parameters determines whether the sign, Γi, of the polarization for nucleus i indicates absorption (A) or emission (E).

Table 1. Result of CIDNP signs analysis of protons (S)-KP-(S)-Trp dyad

Protons	μ	ε	Δ g	α	Y	Sign of CIDNP
Aromatics orto	-	+	+	-	+	A
Indol (23)	-	+	-	-	+	E
CH_2 of Trp (21)	-	+	-	+	+	A
NH (24)	-	+	-	-	+	E

^{1}H CIDNP spectra detected during the photoinduced interaction of KP (racemic) and S-Tryptophan methyl ester in solution are shown in Figure S5.

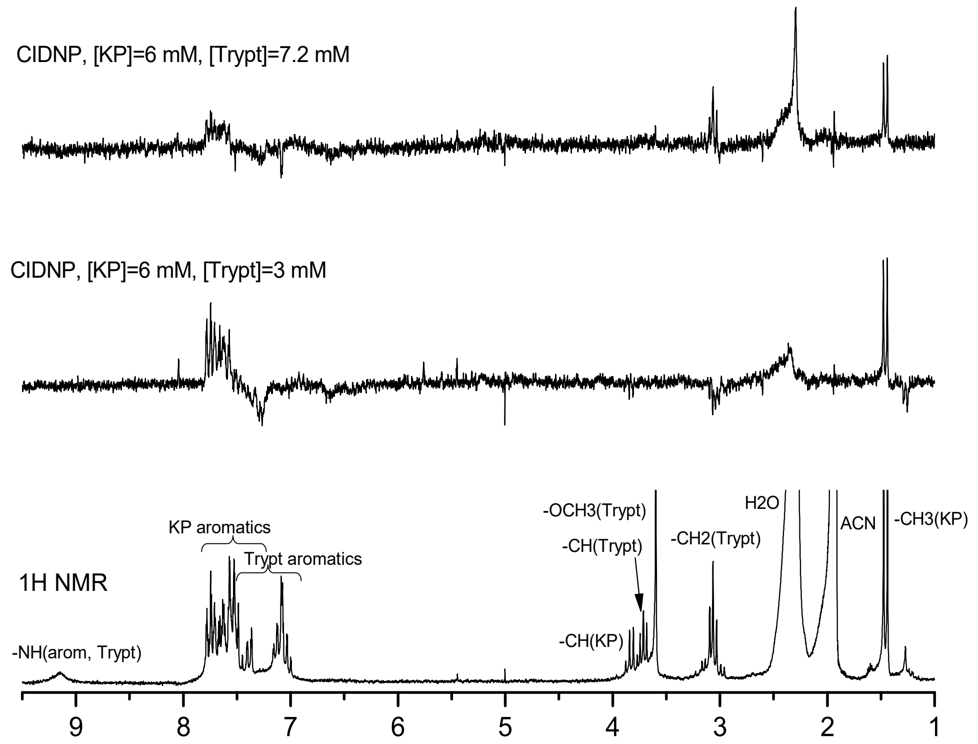

Figure S5. ^{1}H NMR and CIDNP spectra of KP (racemic), 6 mM, and L-Tryptophan methyl ester 3 mM and 7.2 mM, in ACN-d3.

4. *Absorption and fluorescence spectra of the dyad*

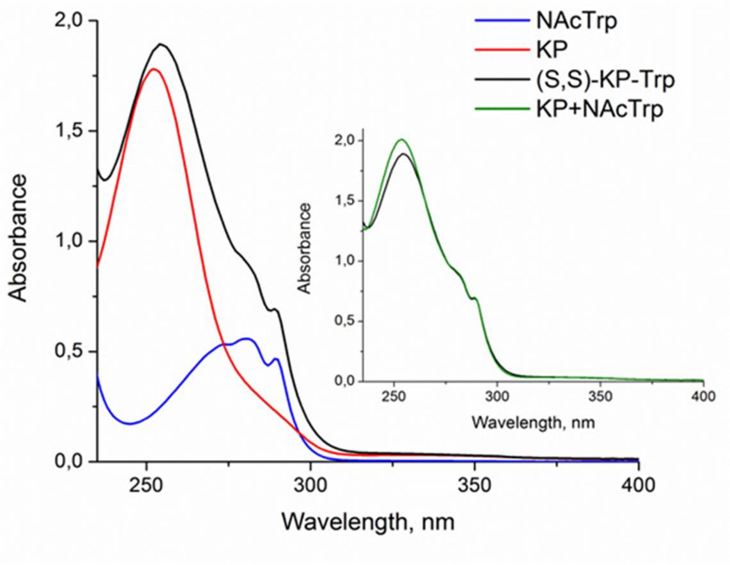

Figure S6. Absorption spectra of parent compounds – (S)-NAcTrp (10^{-4} M) and KP (10^{-4} M) and (S)-KP-(S)-Trp (10^{-4} M) in acetonitrile. The inset shows the summed up spectra of the isolated chromophores and the spectrum of (S,S)-dyad.

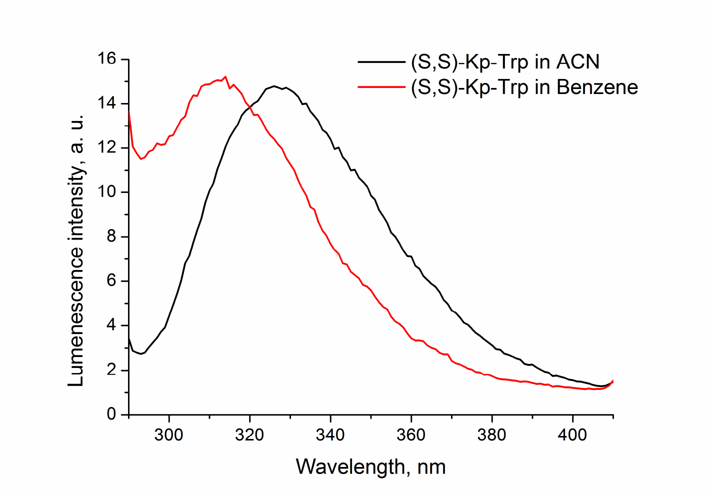

Figure S7. Fluorescence spectra of isoabsorptive solutions of (S)-Kp-(S)-Trp in acetonitrile and benzene at the excitation wavelength of 280 nm in a 1 mm quartz cuvette.

5. *Quantum chemical calculation of various configurations of diastereomers of a dyad (S/R) ketoprofen–(S) tryptophan*

Calculations were performed using software Hyperchem 8 by semi-empirical AM1 method. Energy of dyads spatial configuration was minimized with restriction of selected torsion angle and optimization of all other geometrical parameters.

The pictures presented below show the results of calculations of the dependence of the values of the heat of formation, the distances between donor and acceptor of dyad's diastereomers, and the angle between the planes of the ketoprofen and tryptophan fragments upon rotation of one bond (Figures S8-S10). This is specifically the distances between carbonyl carbon of KP and nitrogen atom of indole fragment of dyad (intercenter distance) and the angles between planes of Trp aromatic ring and "C-CO-C" fragment of KP (interplane angle) calculated for different torsion angle. The rotation was carried out about C -C bond of molecular bridge shown in the Figure S8.

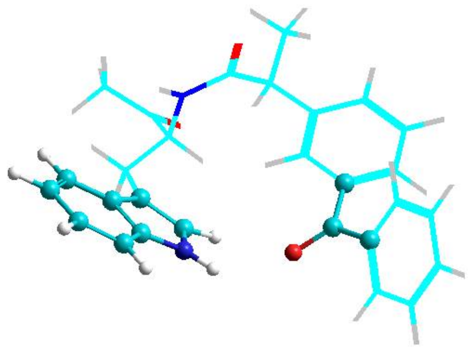

a

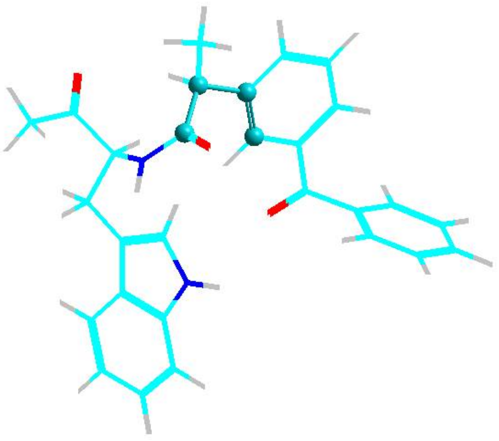

b

Figure S8. KP and Indol fragments (a) and rotating bonds (b) in KP–Trp dyad.

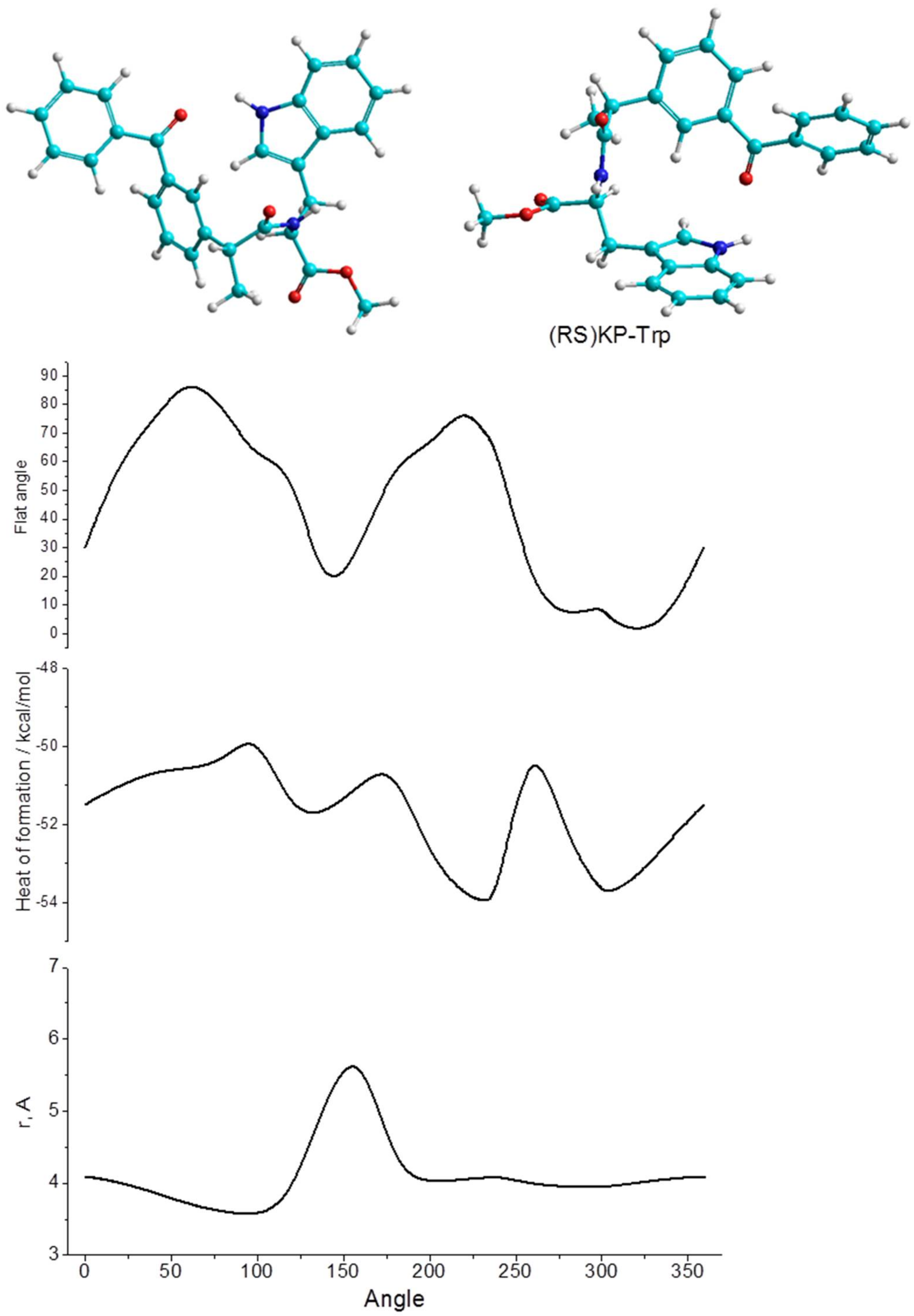

Figure S9. The dependences of: heat of formation (a), interplane angle (b), intercenter distance (c) on the torsion angle values calculated for (R)-KP-(S)-Trp dyad.

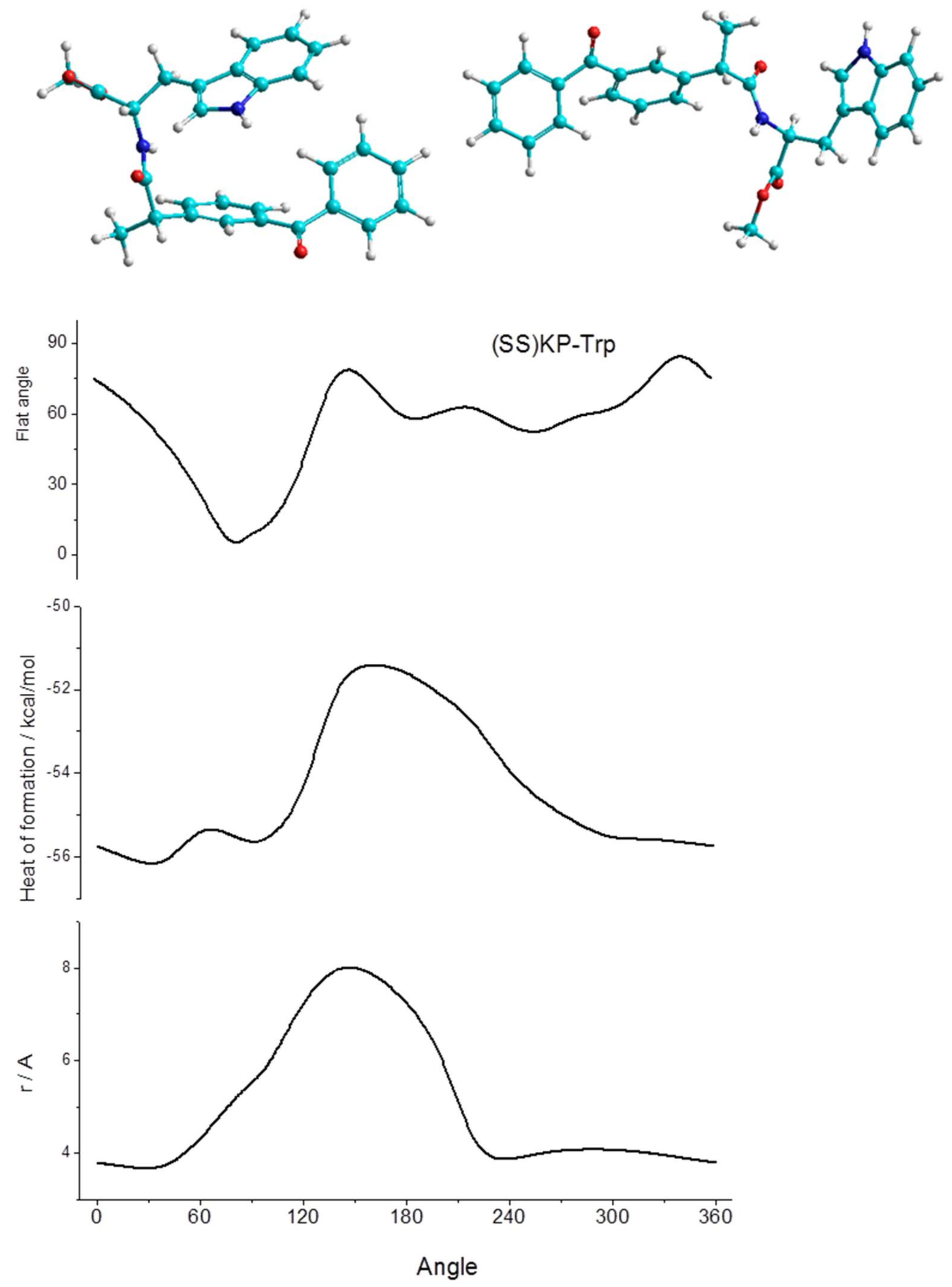

Figure S10. The dependences of: heat of formation (a), interplane angle (b), intercenter distance (c) on the torsion angle values calculated for (SS) KP-(S)Trp dyad.

Moreover, the calculation has shown that in (S,S)-diastereomer, the bulky substituents at the chiral centers are farther apart and do not hinder rotation around the bonds of the molecular bridge (Figure S11).

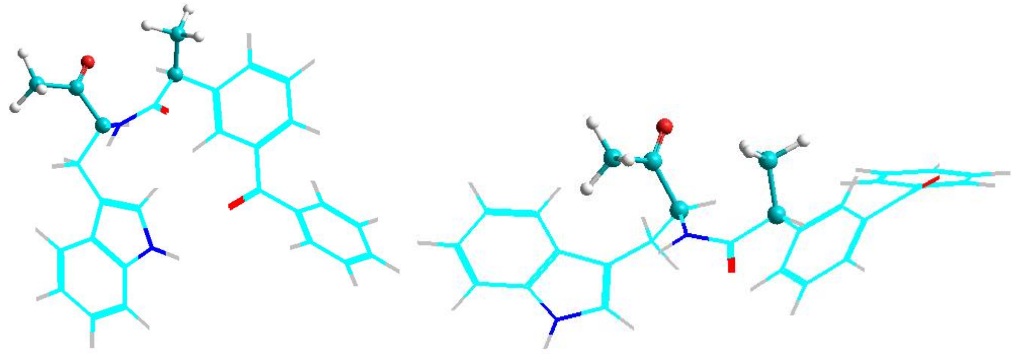

a b

Figure S11. Relative position of substituents at chiral centers for (a) (S,S)- and (b) (R,S)-diastereomers

In general, it is seen that for (R,S)-diastereomer, the rotation about bonds of bridge leads to significantly bigger removal of fragments from each other than for (S,S). It can be assumed that it is due to the arrangement of bulky substituents relative to chiral centers, where they can more or less interfere with each other under rotation (Figure S11).

References

1.F. Savariz, M. Foglio, J. De Carvalho, A. Ruiz, M. Duarte, M. Da Rosa, E. Meyer, M. Sarragiotto. Synthesis and Evaluation of New β-Carboline-3-(4-benzylidene)-4H-oxazol-5-one Derivatives as Antitumor Agents *Molecules.* 2012, 17, 5, pp 6100-6113. https://doi.org/10.3390/molecules17056100]

2. I. Andreu, F. Boscá, L. Sanchez, I. Morera, P. Camps, M.. Miranda. Efficient and Selective Photogeneration of Cholesterol-Derived Radicals by Intramolecular Hydrogen Abstraction in Model Dyads. *Org. Lett.* 2006, 8, 20, pp 4597-4600. https://doi.org/10.1021/ol061854c]

3. Closs, G. L., & Czeropski, M. S. (1977). Amendment of the CIDNP phase rules. Radical pairs leading to triplet states. Journal of the American Chemical Society, 99(18), 6127–6128]
